# *N*-[3a-(4-Bromo­phen­yl)-8b-hy­droxy-6,8-dimeth­oxy-3-phenyl-2,3,3a,8b-tetra­hydro-1*H*-cyclo­penta­[*b*]benzofuran-1-yl]formamide monohydrate

**DOI:** 10.1107/S1600536812049641

**Published:** 2012-12-08

**Authors:** Emmanuel Aubert, Frédéric Thuaud, Nigel Ribeiro, Laurent Désaubry, Enrique Espinosa

**Affiliations:** a Cristallographie, Résonance Magnétique et Modélisations (CRM2), UMR CNRS-UHP 7036, Institut Jean Barriol, Université de Lorraine, BP 70239, Bd des Aiguillettes, 54506 Vandoeuvre-les-Nancy, France; b Therapeutic Innovation Laboratory (UMR7200), Department of Medicinal Chemistry, Faculté de Pharmacie, CNRS Université de Strasbourg, 74 Route du Rhin, BP 60024, 67401 Illkirch, France

## Abstract

In the title compound, C_26_H_24_BrNO_5_·H_2_O, a synthetic analogue of natural flavagline, the cyclo­pentane ring adopts an envelope conformation (the flap atom bearing the phenyl group) and the vicinal phenyl and bromo­phenyl groups are slightly shifted relative to each other [C_Ph_—C—C—C_PhBr_ = 36.3 (2)°]. Intra­molecular N—H⋯O and C—H⋯O hydrogen bonds form *S*(5) motifs. In the crystal, the organic and the water mol­ecules are linked by an O—H⋯O hydrogen bond. Pairs of organic and water mol­ecules, located about inversion centers, inter­act through O—H⋯O hydrogen bonds, forming *R*_4_^4^(20) and *R*_4_^4^(26) motifs, which together lead to *C*_2_^2^(9) motifs. The crystal packing is also characterized by N—H⋯O and C—H⋯O hydrogen bonds between neighbouring organic mol­ecules, forming *R*_2_^2^(10) and *R*_2_^2^(18) motifs, respectively.

## Related literature

For flavaglines and their anti­cancer, neuro- and cardioprotective activities, see: Ribeiro *et al.* (2012*a* ▶,*b* ▶); Bernard *et al.* (2011 ▶); Thuaud *et al.* (2011 ▶). For hydrogen-bond motifs, see: Bernstein *et al.* (1995 ▶).
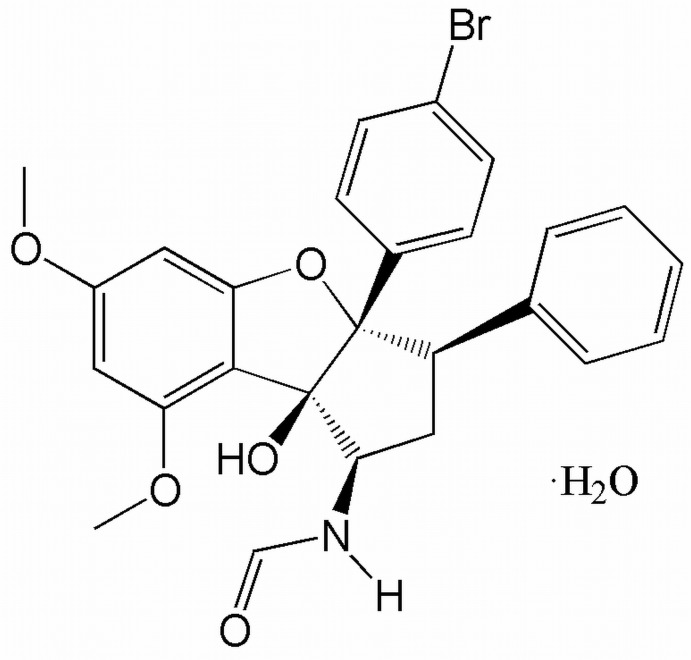


## Experimental

### 

#### Crystal data

C_26_H_24_BrNO_5_·H_2_O
*M* *_r_* = 528.39
Triclinic, 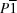

*a* = 8.5941 (2) Å
*b* = 12.1107 (4) Å
*c* = 12.6642 (3) Å
α = 70.537 (2)°
β = 73.495 (2)°
γ = 73.898 (2)°
*V* = 1166.98 (5) Å^3^
*Z* = 2
Cu *K*α radiation
μ = 2.77 mm^−1^
*T* = 110 K
0.34 × 0.26 × 0.07 mm

#### Data collection

Agilent SuperNova diffractometer
Absorption correction: analytical [*CrysAlis PRO* (Agilent, 2012 ▶), based on expressions derived from Clark & Reid (1995 ▶)] *T*_min_ = 0.551, *T*_max_ = 0.868
24473 measured reflections
4860 independent reflections
4814 reflections with *I* > 2σ(*I*)
*R*_int_ = 0.021

#### Refinement

*R*[*F* ^2^ > 2σ(*F* ^2^)] = 0.027
*wR*(*F* ^2^) = 0.074
*S* = 1.05
4860 reflections
322 parameters
3 restraints
H atoms treated by a mixture of independent and constrained refinement
Δρ_max_ = 0.38 e Å^−3^
Δρ_min_ = −0.59 e Å^−3^

### 

Data collection: *CrysAlis PRO* (Agilent, 2012 ▶); cell refinement: *CrysAlis PRO*; data reduction: *CrysAlis PRO*; program(s) used to solve structure: *SIR92* (Altomare *et al.*, 1994 ▶); program(s) used to refine structure: *SHELXL97* (Sheldrick, 2008) ▶; molecular graphics: *Mercury* (Macrae *et al.*, 2006 ▶); software used to prepare material for publication: *WinGX* (Farrugia, 2012) ▶.

## Supplementary Material

Crystal structure: contains datablock(s) I, global. DOI: 10.1107/S1600536812049641/bx2432sup1.cif

Structure factors: contains datablock(s) I. DOI: 10.1107/S1600536812049641/bx2432Isup2.hkl

Supplementary material file. DOI: 10.1107/S1600536812049641/bx2432Isup3.cml

Additional supplementary materials:  crystallographic information; 3D view; checkCIF report

## Figures and Tables

**Table 1:** Hydrogen-bond geometry (Å, °)

*D*—H⋯*A*	*D*—H	H⋯*A*	*D*⋯*A*	*D*—H⋯*A*
N18—H18⋯O17	0.83	2.31	2.652 (2)	106
C32—H32⋯O1	0.95	2.28	2.661 (2)	103
O17—H17⋯O33	0.84	1.90	2.686 (2)	156
N18—H18⋯O17^i^	0.83	2.38	3.185 (2)	163
C28—H28⋯O33^i^	0.95	2.53	3.328 (2)	142
C29—H29⋯O15^i^	0.95	2.62	3.516 (2)	157
O33—H33*A*⋯O13^ii^	0.81	2.21	3.015 (2)	179
O33—H33*B*⋯O20^iii^	0.81	1.90	2.699 (2)	170

Symmetry codes: (i) 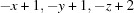; (ii) 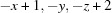; (iii) 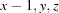.

**Fig. 1.**
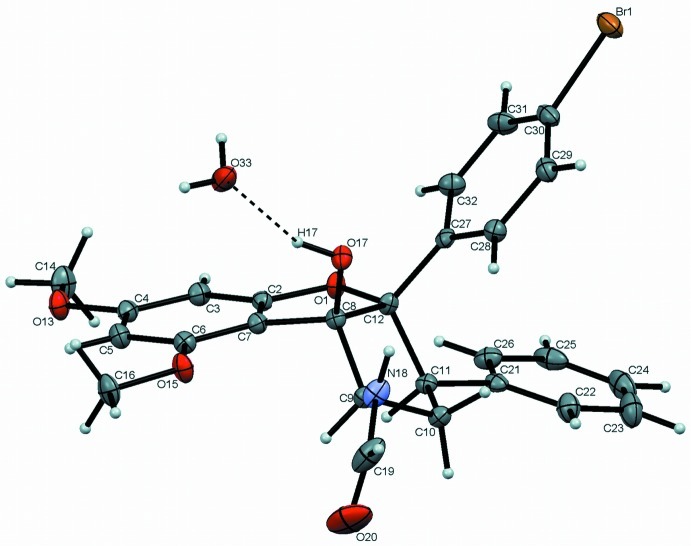
View of the title compound showing the atomic numbering and 50% probability displacement ellipsoids.

## supplementary crystallographic information

### Experimental

Suitable crystals of the title compound were obtained by slow evaporation from acetone at room temperature.

### Refinement

H(—C) hydrogen atoms were postioned geometrically and were treated as riding on their parent C atoms. The torsion angles of the two methyl groups were obtained by refinement. The hydrogen atom of the hydroxyl group was treated as riding on his parent O atom, while the torsion angle of the associated group was refined. Hydrogen atoms of the water molecule and of the formamide groupwere restrained to O—H=0.82 (1) Å and to N—H=0.87 (2) Å, respectively.

### Crystal data

**Table:**

C_26_H_24_BrNO_5_·H_2_O	*Z* = 2
*M**_r_* = 528.39	*F*(000) = 544
Triclinic, *P*1	*D*_x_ = 1.504 Mg m^−^^3^
Hall symbol: -P 1	Cu *K*α radiation, λ = 1.54184 Å
*a* = 8.5941 (2) Å	Cell parameters from 17225 reflections
*b* = 12.1107 (4) Å	θ = 3.8–76.4°
*c* = 12.6642 (3) Å	µ = 2.77 mm^−^^1^
α = 70.537 (2)°	*T* = 110 K
β = 73.495 (2)°	Plate, colourless
γ = 73.898 (2)°	0.34 × 0.26 × 0.07 mm
*V* = 1166.98 (5) Å^3^	

### Data collection

**Table:**

Agilent SuperNova diffractometer	4860 independent reflections
Radiation source: SuperNova (Cu) X-ray Source	4814 reflections with *I* > 2σ(*I*)
Mirror monochromator	*R*_int_ = 0.021
Detector resolution: 10.4508 pixels mm^-1^	θ_max_ = 76.6°, θ_min_ = 3.8°
ω scans	*h* = −10→10
Absorption correction: analytical [*CrysAlis PRO* (Agilent, 2012), based on expressions derived from Clark & Reid (1995)]	*k* = −15→12
*T*_min_ = 0.551, *T*_max_ = 0.868	*l* = −15→15
24473 measured reflections	

### Refinement

**Table:**

Refinement on *F*^2^	Primary atom site location: structure-invariant direct methods
Least-squares matrix: full	Secondary atom site location: difference Fourier map
*R*[*F*^2^ > 2σ(*F*^2^)] = 0.027	Hydrogen site location: difference Fourier map
*wR*(*F*^2^) = 0.074	H atoms treated by a mixture of independent and constrained refinement
*S* = 1.05	*w* = 1/[σ^2^(*F*_o_^2^) + (0.0341*P*)^2^ + 1.1295*P*] where *P* = (*F*_o_^2^ + 2*F*_c_^2^)/3
4860 reflections	(Δ/σ)_max_ = 0.002
322 parameters	Δρ_max_ = 0.38 e Å^−^^3^
3 restraints	Δρ_min_ = −0.59 e Å^−^^3^

### Special details

**Table:**

Geometry. All e.s.d.'s (except the e.s.d. in the dihedral angle between two l.s. planes) are estimated using the full covariance matrix. The cell e.s.d.'s are taken into account individually in the estimation of e.s.d.'s in distances, angles and torsion angles; correlations between e.s.d.'s in cell parameters are only used when they are defined by crystal symmetry. An approximate (isotropic) treatment of cell e.s.d.'s is used for estimating e.s.d.'s involving l.s. planes.
Refinement. Refinement of *F*^2^ against ALL reflections. The weighted *R*-factor *wR* and goodness of fit *S* are based on *F*^2^, conventional *R*-factors *R* are based on *F*, with *F* set to zero for negative *F*^2^. The threshold expression of *F*^2^ > σ(*F*^2^) is used only for calculating *R*-factors(gt) *etc*. and is not relevant to the choice of reflections for refinement. *R*-factors based on *F*^2^ are statistically about twice as large as those based on *F*, and *R*- factors based on ALL data will be even larger.

### Fractional atomic coordinates and isotropic or equivalent isotropic displacement parameters (Å^2^)

**Table:**

	*x*	*y*	*z*	*U*_iso_*/*U*_eq_	
Br1	0.05195 (2)	0.900096 (15)	0.704006 (16)	0.02799 (7)	
O17	0.51357 (13)	0.41085 (9)	0.92476 (9)	0.0149 (2)	
H17	0.4752	0.3496	0.9649	0.022*	
O1	0.60153 (14)	0.39338 (9)	0.65437 (9)	0.0142 (2)	
O13	0.62790 (15)	−0.02946 (10)	0.73217 (10)	0.0197 (2)	
O15	0.73519 (15)	0.13138 (10)	0.99384 (9)	0.0182 (2)	
O33	0.32657 (16)	0.26192 (11)	1.08263 (11)	0.0241 (3)	
N18	0.79574 (19)	0.41272 (13)	0.96990 (11)	0.0197 (3)	
O20	1.05986 (18)	0.32592 (16)	0.98704 (13)	0.0426 (4)	
C21	0.82221 (18)	0.59920 (14)	0.55426 (13)	0.0143 (3)	
C11	0.81098 (18)	0.49342 (13)	0.65978 (12)	0.0128 (3)	
H11	0.8828	0.4215	0.6361	0.015*	
C2	0.62613 (18)	0.27675 (13)	0.71823 (13)	0.0133 (3)	
C6	0.69272 (19)	0.14421 (14)	0.89379 (13)	0.0145 (3)	
C7	0.66121 (18)	0.26081 (13)	0.82267 (12)	0.0131 (3)	
C30	0.2423 (2)	0.77447 (14)	0.69946 (14)	0.0190 (3)	
C29	0.3489 (2)	0.75174 (14)	0.77164 (14)	0.0182 (3)	
H29	0.3348	0.8038	0.8171	0.022*	
C10	0.86995 (19)	0.50048 (14)	0.75986 (12)	0.0141 (3)	
H10A	0.8169	0.5772	0.7788	0.017*	
H10B	0.9918	0.4923	0.7419	0.017*	
C28	0.47664 (19)	0.65176 (14)	0.77651 (13)	0.0153 (3)	
H28	0.5502	0.6353	0.8259	0.018*	
C9	0.81604 (19)	0.39483 (14)	0.85875 (12)	0.0143 (3)	
H9	0.9024	0.3211	0.8529	0.017*	
C27	0.49841 (18)	0.57505 (13)	0.70987 (12)	0.0125 (3)	
C14	0.5949 (2)	−0.01482 (16)	0.62326 (15)	0.0249 (4)	
H14B	0.4881	0.0400	0.6158	0.037*	
H14C	0.5910	−0.0926	0.6173	0.037*	
H14A	0.6829	0.0182	0.5621	0.037*	
C26	0.78129 (19)	0.59676 (16)	0.45604 (13)	0.0190 (3)	
H26	0.7440	0.5295	0.4570	0.023*	
C22	0.8773 (2)	0.69841 (15)	0.55031 (14)	0.0211 (3)	
H22	0.9035	0.7025	0.6167	0.025*	
C8	0.65169 (18)	0.37999 (13)	0.83925 (12)	0.0119 (3)	
C31	0.2653 (2)	0.70372 (16)	0.62875 (14)	0.0213 (3)	
H31	0.1942	0.7225	0.5772	0.026*	
C4	0.64028 (19)	0.07103 (14)	0.75429 (13)	0.0155 (3)	
C12	0.63521 (18)	0.46509 (13)	0.71503 (12)	0.0117 (3)	
C25	0.7943 (2)	0.69131 (17)	0.35686 (14)	0.0239 (4)	
H25	0.7630	0.6894	0.2914	0.029*	
C32	0.3941 (2)	0.60461 (15)	0.63388 (13)	0.0181 (3)	
H32	0.4115	0.5559	0.5846	0.022*	
C3	0.61409 (19)	0.18505 (14)	0.67967 (13)	0.0151 (3)	
H3	0.5896	0.1995	0.6071	0.018*	
C5	0.68119 (19)	0.04909 (14)	0.86002 (13)	0.0166 (3)	
H5	0.7009	−0.0302	0.9083	0.020*	
C16	0.7727 (2)	0.01173 (15)	1.06525 (14)	0.0226 (3)	
H16A	0.6723	−0.0219	1.0945	0.034*	
H16C	0.8136	0.0127	1.1297	0.034*	
H16B	0.8579	−0.0374	1.0204	0.034*	
C19	0.9206 (3)	0.37794 (19)	1.02350 (15)	0.0300 (4)	
H19	0.9008	0.3945	1.0950	0.036*	
C23	0.8944 (3)	0.79191 (16)	0.45014 (16)	0.0289 (4)	
H23	0.9348	0.8582	0.4481	0.035*	
C24	0.8529 (2)	0.78850 (16)	0.35359 (15)	0.0279 (4)	
H24	0.8644	0.8524	0.2854	0.033*	
H33A	0.338 (3)	0.1995 (14)	1.1318 (16)	0.037 (7)*	
H33B	0.244 (2)	0.273 (2)	1.058 (2)	0.045 (7)*	
H18	0.704 (2)	0.447 (2)	0.9998 (19)	0.031 (6)*	

### Atomic displacement parameters (Å^2^)

**Table:**

	*U*^11^	*U*^22^	*U*^33^	*U*^12^	*U*^13^	*U*^23^
Br1	0.02195 (10)	0.01868 (10)	0.03460 (12)	0.00392 (7)	−0.00600 (8)	−0.00272 (8)
O17	0.0187 (5)	0.0133 (5)	0.0112 (5)	−0.0043 (4)	0.0008 (4)	−0.0039 (4)
O1	0.0223 (5)	0.0122 (5)	0.0114 (5)	−0.0053 (4)	−0.0063 (4)	−0.0035 (4)
O13	0.0288 (6)	0.0137 (5)	0.0201 (6)	−0.0040 (5)	−0.0077 (5)	−0.0074 (4)
O15	0.0281 (6)	0.0134 (5)	0.0139 (5)	−0.0020 (4)	−0.0102 (4)	−0.0017 (4)
O33	0.0224 (6)	0.0228 (6)	0.0261 (6)	−0.0097 (5)	−0.0116 (5)	0.0040 (5)
N18	0.0255 (7)	0.0251 (7)	0.0121 (6)	−0.0115 (6)	−0.0061 (5)	−0.0028 (5)
O20	0.0302 (8)	0.0681 (11)	0.0305 (7)	−0.0182 (7)	−0.0182 (6)	0.0016 (7)
C21	0.0110 (6)	0.0166 (7)	0.0120 (7)	−0.0010 (5)	−0.0001 (5)	−0.0029 (6)
C11	0.0126 (7)	0.0144 (7)	0.0105 (6)	−0.0026 (5)	−0.0016 (5)	−0.0033 (5)
C2	0.0130 (7)	0.0124 (7)	0.0135 (7)	−0.0023 (5)	−0.0017 (5)	−0.0036 (5)
C6	0.0153 (7)	0.0149 (7)	0.0128 (7)	−0.0024 (6)	−0.0027 (5)	−0.0040 (6)
C7	0.0135 (7)	0.0136 (7)	0.0129 (7)	−0.0028 (5)	−0.0024 (5)	−0.0049 (5)
C30	0.0154 (7)	0.0152 (7)	0.0184 (7)	0.0008 (6)	−0.0012 (6)	0.0004 (6)
C29	0.0196 (8)	0.0149 (7)	0.0178 (7)	−0.0021 (6)	−0.0021 (6)	−0.0043 (6)
C10	0.0146 (7)	0.0162 (7)	0.0121 (7)	−0.0048 (6)	−0.0035 (5)	−0.0028 (6)
C28	0.0165 (7)	0.0157 (7)	0.0140 (7)	−0.0035 (6)	−0.0039 (5)	−0.0037 (6)
C9	0.0161 (7)	0.0160 (7)	0.0122 (7)	−0.0043 (6)	−0.0057 (5)	−0.0027 (6)
C27	0.0122 (7)	0.0135 (7)	0.0104 (6)	−0.0049 (5)	−0.0010 (5)	−0.0009 (5)
C14	0.0386 (10)	0.0212 (8)	0.0214 (8)	−0.0075 (7)	−0.0091 (7)	−0.0107 (7)
C26	0.0159 (7)	0.0253 (8)	0.0150 (7)	−0.0049 (6)	−0.0029 (6)	−0.0041 (6)
C22	0.0267 (8)	0.0179 (8)	0.0169 (7)	−0.0052 (6)	−0.0034 (6)	−0.0030 (6)
C8	0.0136 (7)	0.0125 (7)	0.0093 (6)	−0.0027 (5)	−0.0020 (5)	−0.0027 (5)
C31	0.0171 (7)	0.0267 (9)	0.0178 (7)	−0.0013 (6)	−0.0071 (6)	−0.0030 (6)
C4	0.0150 (7)	0.0145 (7)	0.0183 (7)	−0.0026 (6)	−0.0013 (6)	−0.0086 (6)
C12	0.0148 (7)	0.0130 (7)	0.0098 (6)	−0.0042 (5)	−0.0037 (5)	−0.0043 (5)
C25	0.0169 (8)	0.0335 (10)	0.0138 (7)	0.0015 (7)	−0.0043 (6)	−0.0013 (7)
C32	0.0178 (7)	0.0221 (8)	0.0158 (7)	−0.0035 (6)	−0.0054 (6)	−0.0060 (6)
C3	0.0162 (7)	0.0168 (7)	0.0141 (7)	−0.0034 (6)	−0.0030 (5)	−0.0067 (6)
C5	0.0189 (7)	0.0130 (7)	0.0168 (7)	−0.0017 (6)	−0.0041 (6)	−0.0038 (6)
C16	0.0334 (9)	0.0146 (8)	0.0174 (7)	−0.0015 (7)	−0.0107 (7)	0.0005 (6)
C19	0.0378 (11)	0.0429 (11)	0.0155 (8)	−0.0274 (9)	−0.0119 (7)	0.0050 (7)
C23	0.0388 (10)	0.0170 (8)	0.0252 (9)	−0.0075 (7)	−0.0039 (8)	0.0005 (7)
C24	0.0293 (9)	0.0210 (8)	0.0193 (8)	0.0017 (7)	−0.0030 (7)	0.0048 (7)

### Geometric parameters (Å, º)

**Table:**

Br1—C30	1.9023 (16)	C10—H10B	0.9900
O17—C8	1.4194 (17)	C28—C27	1.395 (2)
O17—H17	0.8400	C28—H28	0.9500
O1—C2	1.3653 (18)	C9—C8	1.567 (2)
O1—C12	1.4623 (16)	C9—H9	1.0000
O13—C4	1.3737 (18)	C27—C32	1.393 (2)
O13—C14	1.4304 (19)	C27—C12	1.509 (2)
O15—C6	1.3649 (18)	C14—H14B	0.9800
O15—C16	1.4335 (19)	C14—H14C	0.9800
O33—H33A	0.805 (10)	C14—H14A	0.9800
O33—H33B	0.808 (10)	C26—C25	1.391 (2)
N18—C19	1.328 (2)	C26—H26	0.9500
N18—C9	1.4497 (19)	C22—C23	1.394 (2)
N18—H18	0.830 (16)	C22—H22	0.9500
O20—C19	1.226 (3)	C8—C12	1.5882 (19)
C21—C22	1.390 (2)	C31—C32	1.386 (2)
C21—C26	1.397 (2)	C31—H31	0.9500
C21—C11	1.514 (2)	C4—C3	1.389 (2)
C11—C10	1.5270 (19)	C4—C5	1.402 (2)
C11—C12	1.554 (2)	C25—C24	1.387 (3)
C11—H11	1.0000	C25—H25	0.9500
C2—C7	1.379 (2)	C32—H32	0.9500
C2—C3	1.391 (2)	C3—H3	0.9500
C6—C5	1.391 (2)	C5—H5	0.9500
C6—C7	1.399 (2)	C16—H16A	0.9800
C7—C8	1.5035 (19)	C16—H16C	0.9800
C30—C31	1.375 (2)	C16—H16B	0.9800
C30—C29	1.385 (2)	C19—H19	0.9500
C29—C28	1.388 (2)	C23—C24	1.384 (3)
C29—H29	0.9500	C23—H23	0.9500
C10—C9	1.533 (2)	C24—H24	0.9500
C10—H10A	0.9900		
			
C8—O17—H17	109.5	H14B—C14—H14A	109.5
C2—O1—C12	108.14 (11)	H14C—C14—H14A	109.5
C4—O13—C14	117.21 (13)	C25—C26—C21	121.01 (16)
C6—O15—C16	116.71 (12)	C25—C26—H26	119.5
H33A—O33—H33B	112 (3)	C21—C26—H26	119.5
C19—N18—C9	121.62 (16)	C21—C22—C23	120.81 (16)
C19—N18—H18	119.6 (16)	C21—C22—H22	119.6
C9—N18—H18	118.7 (17)	C23—C22—H22	119.6
C22—C21—C26	118.30 (14)	O17—C8—C7	112.92 (12)
C22—C21—C11	122.00 (14)	O17—C8—C9	111.29 (11)
C26—C21—C11	119.67 (14)	C7—C8—C9	114.22 (12)
C21—C11—C10	115.85 (12)	O17—C8—C12	111.92 (11)
C21—C11—C12	115.75 (12)	C7—C8—C12	100.43 (11)
C10—C11—C12	103.66 (11)	C9—C8—C12	105.31 (11)
C21—C11—H11	107.0	C30—C31—C32	118.93 (15)
C10—C11—H11	107.0	C30—C31—H31	120.5
C12—C11—H11	107.0	C32—C31—H31	120.5
O1—C2—C7	113.50 (13)	O13—C4—C3	123.59 (14)
O1—C2—C3	121.92 (13)	O13—C4—C5	114.11 (14)
C7—C2—C3	124.57 (14)	C3—C4—C5	122.30 (14)
O15—C6—C5	123.77 (14)	O1—C12—C27	107.13 (11)
O15—C6—C7	116.53 (13)	O1—C12—C11	109.18 (11)
C5—C6—C7	119.69 (14)	C27—C12—C11	113.61 (12)
C2—C7—C6	118.19 (13)	O1—C12—C8	106.45 (11)
C2—C7—C8	110.01 (13)	C27—C12—C8	116.28 (11)
C6—C7—C8	131.76 (13)	C11—C12—C8	103.86 (11)
C31—C30—C29	121.48 (15)	C24—C25—C26	119.93 (16)
C31—C30—Br1	118.73 (12)	C24—C25—H25	120.0
C29—C30—Br1	119.69 (12)	C26—C25—H25	120.0
C30—C29—C28	118.95 (15)	C31—C32—C27	121.27 (15)
C30—C29—H29	120.5	C31—C32—H32	119.4
C28—C29—H29	120.5	C27—C32—H32	119.4
C11—C10—C9	103.86 (12)	C4—C3—C2	115.59 (14)
C11—C10—H10A	111.0	C4—C3—H3	122.2
C9—C10—H10A	111.0	C2—C3—H3	122.2
C11—C10—H10B	111.0	C6—C5—C4	119.58 (14)
C9—C10—H10B	111.0	C6—C5—H5	120.2
H10A—C10—H10B	109.0	C4—C5—H5	120.2
C29—C28—C27	120.88 (14)	O15—C16—H16A	109.5
C29—C28—H28	119.6	O15—C16—H16C	109.5
C27—C28—H28	119.6	H16A—C16—H16C	109.5
N18—C9—C10	112.30 (12)	O15—C16—H16B	109.5
N18—C9—C8	112.54 (12)	H16A—C16—H16B	109.5
C10—C9—C8	105.93 (11)	H16C—C16—H16B	109.5
N18—C9—H9	108.6	O20—C19—N18	124.46 (18)
C10—C9—H9	108.6	O20—C19—H19	117.8
C8—C9—H9	108.6	N18—C19—H19	117.8
C32—C27—C28	118.33 (14)	C24—C23—C22	120.25 (17)
C32—C27—C12	119.93 (13)	C24—C23—H23	119.9
C28—C27—C12	121.69 (13)	C22—C23—H23	119.9
O13—C14—H14B	109.5	C23—C24—C25	119.66 (16)
O13—C14—H14C	109.5	C23—C24—H24	120.2
H14B—C14—H14C	109.5	C25—C24—H24	120.2
O13—C14—H14A	109.5		
			
C22—C21—C11—C10	−2.2 (2)	Br1—C30—C31—C32	−173.36 (12)
C26—C21—C11—C10	175.78 (13)	C14—O13—C4—C3	3.5 (2)
C22—C21—C11—C12	119.46 (16)	C14—O13—C4—C5	−176.88 (14)
C26—C21—C11—C12	−62.55 (18)	C2—O1—C12—C27	−135.71 (12)
C12—O1—C2—C7	4.89 (16)	C2—O1—C12—C11	100.86 (13)
C12—O1—C2—C3	−175.84 (13)	C2—O1—C12—C8	−10.68 (14)
C16—O15—C6—C5	1.3 (2)	C32—C27—C12—O1	−14.13 (18)
C16—O15—C6—C7	−178.07 (14)	C28—C27—C12—O1	168.42 (13)
O1—C2—C7—C6	−178.42 (13)	C32—C27—C12—C11	106.53 (15)
C3—C2—C7—C6	2.3 (2)	C28—C27—C12—C11	−70.93 (17)
O1—C2—C7—C8	3.52 (18)	C32—C27—C12—C8	−132.98 (14)
C3—C2—C7—C8	−175.74 (14)	C28—C27—C12—C8	49.56 (19)
O15—C6—C7—C2	176.78 (13)	C21—C11—C12—O1	83.18 (15)
C5—C6—C7—C2	−2.7 (2)	C10—C11—C12—O1	−148.84 (11)
O15—C6—C7—C8	−5.7 (2)	C21—C11—C12—C27	−36.31 (17)
C5—C6—C7—C8	174.89 (15)	C10—C11—C12—C27	91.67 (14)
C31—C30—C29—C28	−3.3 (2)	C21—C11—C12—C8	−163.58 (12)
Br1—C30—C29—C28	172.89 (12)	C10—C11—C12—C8	−35.60 (14)
C21—C11—C10—C9	170.38 (12)	O17—C8—C12—O1	−108.19 (12)
C12—C11—C10—C9	42.46 (14)	C7—C8—C12—O1	11.87 (14)
C30—C29—C28—C27	0.1 (2)	C9—C8—C12—O1	130.74 (12)
C19—N18—C9—C10	−89.55 (18)	O17—C8—C12—C27	11.03 (17)
C19—N18—C9—C8	151.02 (15)	C7—C8—C12—C27	131.09 (12)
C11—C10—C9—N18	−155.59 (13)	C9—C8—C12—C27	−110.03 (13)
C11—C10—C9—C8	−32.37 (15)	O17—C8—C12—C11	136.61 (12)
C29—C28—C27—C32	3.3 (2)	C7—C8—C12—C11	−103.33 (12)
C29—C28—C27—C12	−179.23 (14)	C9—C8—C12—C11	15.55 (14)
C22—C21—C26—C25	−0.3 (2)	C21—C26—C25—C24	1.8 (3)
C11—C21—C26—C25	−178.41 (14)	C30—C31—C32—C27	0.7 (2)
C26—C21—C22—C23	−1.3 (2)	C28—C27—C32—C31	−3.7 (2)
C11—C21—C22—C23	176.71 (16)	C12—C27—C32—C31	178.72 (14)
C2—C7—C8—O17	109.96 (14)	O13—C4—C3—C2	177.76 (14)
C6—C7—C8—O17	−67.8 (2)	C5—C4—C3—C2	−1.9 (2)
C2—C7—C8—C9	−121.54 (14)	O1—C2—C3—C4	−179.26 (13)
C6—C7—C8—C9	60.7 (2)	C7—C2—C3—C4	−0.1 (2)
C2—C7—C8—C12	−9.38 (15)	O15—C6—C5—C4	−178.54 (14)
C6—C7—C8—C12	172.91 (16)	C7—C6—C5—C4	0.9 (2)
N18—C9—C8—O17	11.60 (17)	O13—C4—C5—C6	−178.16 (14)
C10—C9—C8—O17	−111.47 (13)	C3—C4—C5—C6	1.5 (2)
N18—C9—C8—C7	−117.72 (14)	C9—N18—C19—O20	−2.1 (3)
C10—C9—C8—C7	119.21 (13)	C21—C22—C23—C24	1.6 (3)
N18—C9—C8—C12	133.08 (13)	C22—C23—C24—C25	−0.1 (3)
C10—C9—C8—C12	10.01 (15)	C26—C25—C24—C23	−1.5 (3)
C29—C30—C31—C32	2.8 (2)		

### Hydrogen-bond geometry (Å, º)

**Table:**

*D*—H···*A*	*D*—H	H···*A*	*D*···*A*	*D*—H···*A*
N18—H18···O17	0.83	2.31	2.652 (2)	106
C32—H32···O1	0.95	2.28	2.661 (2)	103
O17—H17···O33	0.84	1.90	2.686 (2)	156
N18—H18···O17^i^	0.83	2.38	3.185 (2)	163
C28—H28···O33^i^	0.95	2.53	3.328 (2)	142
C29—H29···O15^i^	0.95	2.62	3.516 (2)	157
O33—H33*A*···O13^ii^	0.81	2.21	3.015 (2)	179
O33—H33*B*···O20^iii^	0.81	1.90	2.699 (2)	170

Symmetry codes: (i) −*x*+1, −*y*+1, −*z*+2; (ii) −*x*+1, −*y*, −*z*+2; (iii) *x*−1, *y*, *z*.
